# Epigenetic Suppression of Mouse *Per2* Expression in the Suprachiasmatic Nucleus by the Inhalational Anesthetic, Sevoflurane

**DOI:** 10.1371/journal.pone.0087319

**Published:** 2014-01-31

**Authors:** Keisuke Mori, Norio Iijima, Shimpei Higo, Satoko Aikawa, Izumi Matsuo, Ken Takumi, Atsuhiro Sakamoto, Hitoshi Ozawa

**Affiliations:** 1 Department of Anatomy and Neurobiology, Graduate School of Medicine, Nippon Medical School, Tokyo, Japan; 2 Department of Anesthesiology and Pain Medicine, Graduate School of Medicine, Nippon Medical School, Tokyo, Japan; Pohang University of Science and Technology (POSTECH), Republic of Korea

## Abstract

**Background:**

We previously reported that sevoflurane anesthesia reversibly suppresses the expression of the clock gene, *Period2* (*Per2*), in the mouse suprachiasmatic nucleus (SCN). However, the molecular mechanisms underlying this suppression remain unclear. In this study, we examined the possibility that sevoflurane suppresses *Per2* expression *via* epigenetic modification of the *Per2* promoter.

**Methods:**

Mice were anesthetized with a gas mixture of 2.5% sevoflurane/40% oxygen at a 6 L/min flow for 1 or 4 h. After termination, brains were removed and samples of SCN tissue were derived from frozen brain sections. Chromatin immunoprecipitation (ChIP) assays using anti-acetylated-histone antibodies were performed to investigate the effects of sevoflurane on histone acetylation of the *Per2* promoter. Interaction between the E’-box (a cis-element in the *Per2* promoter) and CLOCK (the *Clock* gene product) was also assessed by a ChIP assay using an anti-CLOCK antibody. The SCN concentration of nicotinamide adenine dinucleotide (NAD^+^), a CLOCK regulator, was assessed by liquid chromatography-mass spectrometry.

**Results:**

Acetylation of histone H4 in the proximal region of the *Per2* promoter was significantly reduced by sevoflurane. This change in the epigenetic profile of the *Per2* gene was observed prior to suppression of *Per2* expression. Simultaneously, a reduction in the CLOCK-E’-box interaction in the *Per2* promoter was observed. Sevoflurane treatment did not affect the concentration of NAD^+^ in the SCN.

**Conclusions:**

Independent of NAD^+^ concentration in the SCN, sevoflurane decreases CLOCK binding to the *Per2* promoter E’-box motif, reducing histone acetylation and leading to suppression of *Per2* expression.

## Introduction

General anesthesia has been used for over 150 years in surgical procedures, and safety improvements have increased its clinical acceptability [1]–[3]. However, much remains unknown about the essence of general anesthesia, especially the molecular events induced by anesthetic agents, and this subject requires further investigation.

In previous studies, we revealed that the inhalational anesthetic, sevoflurane, suppressed the brain expression of *Period2* (*Per2*), a component of the ‘core loop’ of the circadian clock [4]–[6], most notably in the suprachiasmatic nucleus (SCN), the central pacemaker of the circadian rhythm. Quantitative *in situ* hybridization revealed that sevoflurane anesthesia reversibly suppressed *Per2* expression in the SCN of rats and mice [7], [8]. Application of sevoflurane to an SCN slice culture taken from transgenic rats expressing a mouse *Per2* promoter-destabilized luciferase (*Per2-dLuc*) revealed that suppression of bioluminescence led to a phase delay in the bioluminescence rhythm. These results indicated that sevoflurane treatment disturbs the circadian clock, including the *Per2* expression mechanism. However, the molecular mechanism underlying this suppression of *Per2* expression by sevoflurane is still unknown.

The aim of this study was to elucidate the molecular mechanism of *Per2* expression regulation by sevoflurane. We focused on the epigenetic effect of sevoflurane on the *Per2* gene. Epigenetic mechanisms such as histone modification and DNA methylation play important roles in the regulation of gene expression [9], [10]. Histone acetylation, a well-studied histone modification, is a critical feature of circadian expression of clock genes (e.g., *Per2*) and is involved in the induction of *Per2* by exogenous factors such as light [11]. Expression of the *Per2* gene is controlled by a regulatory mechanism consisting of various cis-elements (E’-box, E’’-box, and D-box in the proximal region of the promoter and cyclic-AMP responsive element (CRE) in the distal region of promoter). Binding of a CLOCK/BMAL1 complex (a product of two clock genes) to the E’-box induces *Per2* expression *via* the acetylation of adjacent histones by the histone acetyltransferase (HAT) activity of CLOCK [12]. This HAT activity is counterbalanced by SIRT1, the product of another clock gene that has nicotinamide adenine dinucleotide (NAD^+^)-dependent histone deacetylase (HDAC) activity, binds to the CLOCK/BMAL1 complex to form a heterotrimer [13], [14].

In this study, we first examined the effects of sevoflurane on DNA methylation and histone acetylation in the *Per2* promoter. We then measured the binding of the CLOCK/BMAL1 complex to the E’-box of the *Per2* promoter in sevoflurane-treated mice compared with controls. We also used liquid chromatography-mass spectrometry (LC-MS) to assess the SCN concentration of NAD^+^ and its metabolites, which regulate SIRT1 and CLOCK.

## Materials and Methods

### Animals

Male C57BL/6J mice (Clea Japan Inc., Tokyo, Japan), aged 8–10 weeks, were used in all experiments. Mice were housed for at least 2 weeks for adaptation to a standard 14 h light/10 h dark cycle (lights on from 06∶00 to 20∶00) with food and water *ad libitum*. All experiments in this study were carried out according to the National Institute of Health Guidelines for the Care and Use of Laboratory Animals, and were approved by the Committee for Animal Research in Nippon Medical School.

### Condition of Anesthesia

Mice were transferred to constant dark conditions for 12 h prior to anesthetic treatment. The mice were then placed in a light-proof chamber (36×24×14 cm) and exposed to a gas mixture of 2.5% sevoflurane (Maruishi Pharmaceutical, Osaka, Japan) and 40% oxygen at a flow rate of 6 L/min. Transfer of mice between the cage and the anesthesia chamber was performed under dim red light. Sevoflurane application began at 08∶00 because our previous study showed that morning anesthesia (08∶00–12∶00) was most effective at suppressing *Per2* expression [15]. The mice were anesthetized for 1 h (sampled at 09∶00), and 4 h (sampled at 12∶00). To maintain normal body temperature, the chamber was placed on a heated sheet. In this anesthetic condition, we confirmed that the body temperature and O_2_ saturation were kept constant and mice regained responsiveness to handling a few minutes after cessation of anesthesia [7].

### SCN Tissue Sampling

Each mouse was terminated by momentary cervical dislocation under dim red light, and their brains were removed and snap frozen in pre-chilled hexane at −80°C. Two consecutive coronal sections containing the SCN (300 µm thick, from approximately −0.90 to −0.30 mm from the bregma) were cut using a cryostat (Leica 3050; Leica Microsystems GmbH, Watzlar Germany). Sections were placed on a glass slide and the SCN dissected using a microdissection punch (φ 1.0 mm; Kai Industries, Gifu, Japan) at −20°C under a stereomicroscope (Fig. S1). Two dissected samples containing the SCN were collected from each mouse and stored at −80°C until use.

### Quantitative Real-time Polymerase Chain Reaction (PCR)

Total RNA was isolated from the dissected SCN samples (n = 5 for each group) using a spin column-based kit (NucleoSpin RNA XS; Macherey-Nagel, D
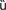
ren, Germany). The purified RNA was reverse transcribed to cDNA using PrimeScript reverse transcriptase (Takara Bio, Shiga, Japan). Synthesized cDNA equivalent to 20 ng total RNA was used as a PCR template. We quantified the expression of *Per2* and *Hsp90ab1* (as an internal control) by real-time PCR, using the SYBR premix EX taq II reagent and a TP 3500 thermal cycler (Takara Bio). We confirmed that sevoflurane treatment had no significant effect on the expression of *Hsp90ab1* in the SCN (Fig. S2). The PCR primers were: mPer2-F, 5′-CTA CCT GGT CAA GGT GCA AGA G-3′; mPer2-R, 5′-TTG GTG TGT GGG TTG TTG TG-3′; hsp90ab1-F, 5′-ATG TCC CTC ATC ATC AAC ACT TTC-3′; hsp90ab1-R, 5′-AGG CTC TCA TAT CGA ATC TTG TCC-3′. The amplification program was: one cycle of 95°C/30 s followed by 40 cycles of 95°C/5 s and 60°C/30 s. Results are presented as a ratio of *Per2* copy number/*Hsp90ab1* copy number.

### Methylation Assay

We assessed the effect of the sevoflurane on methylation profile of the *Per2* promoter by means of bisulfite sequencing. Mice anesthetized with sevoflurane for 4 h (n = 4) and non-anesthetized controls (n = 4) were used for DNA methylation analysis. Genomic DNA was isolated from dissected SCN samples using a NucleoSpin Tissue XS kit (Macherey-Nagel). Isolated DNA was bisulfite converted and purified using the MethylEasy Xceed Kit (Human Genetic Signatures, Randwick, NSW, Australia). Verification of the bisulfite conversion was performed using a positive control supplied in the MethylEasy Xceed Kit (Fig. S3). Bisulfite-converted *Per2* promoter region containing the E’-box sequence was PCR amplified using EpiTaq HS polymerase (Takara Bio) with the following strand-specific primers: Forward, 5′-TTA GTA TGT AAA TGA GGT GGT ATT T-3′; reverse, 5′-AAA CCC CAA CTC CAA CTA TCC-3′. The amplification program was as follows: one cycle of 98°C/30 s, followed by 30 cycles of 98°C/10 s, 57°C/30 s, and 72°C/30 s. The PCR product was subcloned into a pGEM-T easy vector (Promega, Madison WI, USA) and transformed. Plasmids from a random selection of 24 clones per animal (96 clones per group) were purified and sequenced. Only one clone from a non-sevoflurane-treated control was excluded from the data because the sequence of the plasmid from that clone was different from the predicted sequence of the PCR amplification. The amount of methylation of individual CpG sites in the *Per2* promoter was averaged per animal per group, and the percentage of methylated cytosine was calculated.

### Chromatin Immunoprecipitation (ChIP) Assay

ChIP assays were carried out using Magna ChIP G tissue kit (Merck Millipore, Darmstadt, Germany), broadly following the protocol supplied by the manufacturer with slight modifications. The dissected SCN tissues (n = 6) were fixed with 1% formaldehyde for 10 min at 25°C, transferred into 500 µL of lysis buffer (0.5% SDS, 10 mM EDTA, 50 mM Tris-HCl [pH 8.0]) and sonicated for 10 min (pulsed 10 times for 30 s, paused for 30 s; 250 W power) in a Bioruptor 250 (Tosho Denki, Yokohama, Japan). Cross-linked chromatin was immunoprecipitated with an anti-acetylated histone H3 (Lys9) antibody (#17-609; Merck Millipore), an anti-acetylated histone H4 (Lys16) antibody (#17-10101; Merck Millipore), or an anti-CLOCK antibody (ab3517; Abcam, Cambridge, UK). Immunoprecipitated DNA was analyzed by semi-quantitative PCR according to Naruse *et al*. [11] with slight modification. The proximal region of the *Per2* promoter containing the E’-box, E’’-box, and D-box (−252/+27 base pair from TSS) was amplified with the following primers: mPer2-prox-F, 5′-AAG TGG ACG AGC CTA CTC GC-3′; mPer2-prox-R, 5′-AGC GCC GCT GCC GCC GCG TC-3′. The distal region of the *Per2* promoter containing CRE (−1634/−1444) was amplified with the following primers: mPer2-distal-F, 5′-GAC TCT GCC AGG TGG ATG AG-3′; mPer2-distal-R, 5′-CAG CAC CTC TGG TTC CTC TG-3′. The PCR program was: one cycle of 95°C/5 min; 28–32 cycles (non-saturated conditions) of 94°C/30 s; 62°C/30 s; 72°C/30 s for the distal region, 29–34 cycles (non-saturated conditions) of 95°C/30 s; 68°C/60 s for the proximal region. Aliquots of chromatin obtained before immunoprecipitation were also analyzed (Input DNA). For semi-quantitative analysis, the PCR products were separated on a 2% agarose gel, stained with ethidium bromide, and analyzed with a fluorescence gel scanner (Benchtop UV Transilluminator; UVP LLC, Upland, CA, USA) and Image J software. Experiments were repeated at least twice to confirm reproducibility.

### LC-MS Quantification of NAD^+^ and Metabolites

The concentration of the three main metabolites of the NAD^+^ salvage pathway (NAD^+^, nicotinamide [NAM], and nicotinamide mononucleotide [NMN]) in the dissected SCNs was assessed using LC-MS, according to Yamada *et al*. [16] with slight modification. A detailed protocol for LC-MS quantification is described in the Text_S1. The protein content in these samples was assayed using the Bio-Rad Protein Assay (Bio-Rad, Hecules, CA, USA) and used to normalize the data obtained from LC-MS in each sample.

### Statistical Analysis

For the expression analysis of *Per2* and the ChIP assays using anti-acetylated histone antibodies, two-way analysis of variance (ANOVA) followed by Bonferroni tests were applied to determine the effects of sevoflurane anesthesia over time. For the ChIP assay using an anti-CLOCK antibody and the quantification of NAD^+^ and its metabolites, a Student’s t-test was applied to determine the statistical difference between sevoflurane treated and non-anesthetized mice. All statistical analyses were performed using IBM SPSS statistics software. A *p*-value of less than 0.05 (*p*<0.05) was considered statistically significant.

## Results

### Sevoflurane Suppresses the Expression of *Per2* mRNA in the SCN

**Figure 1:**
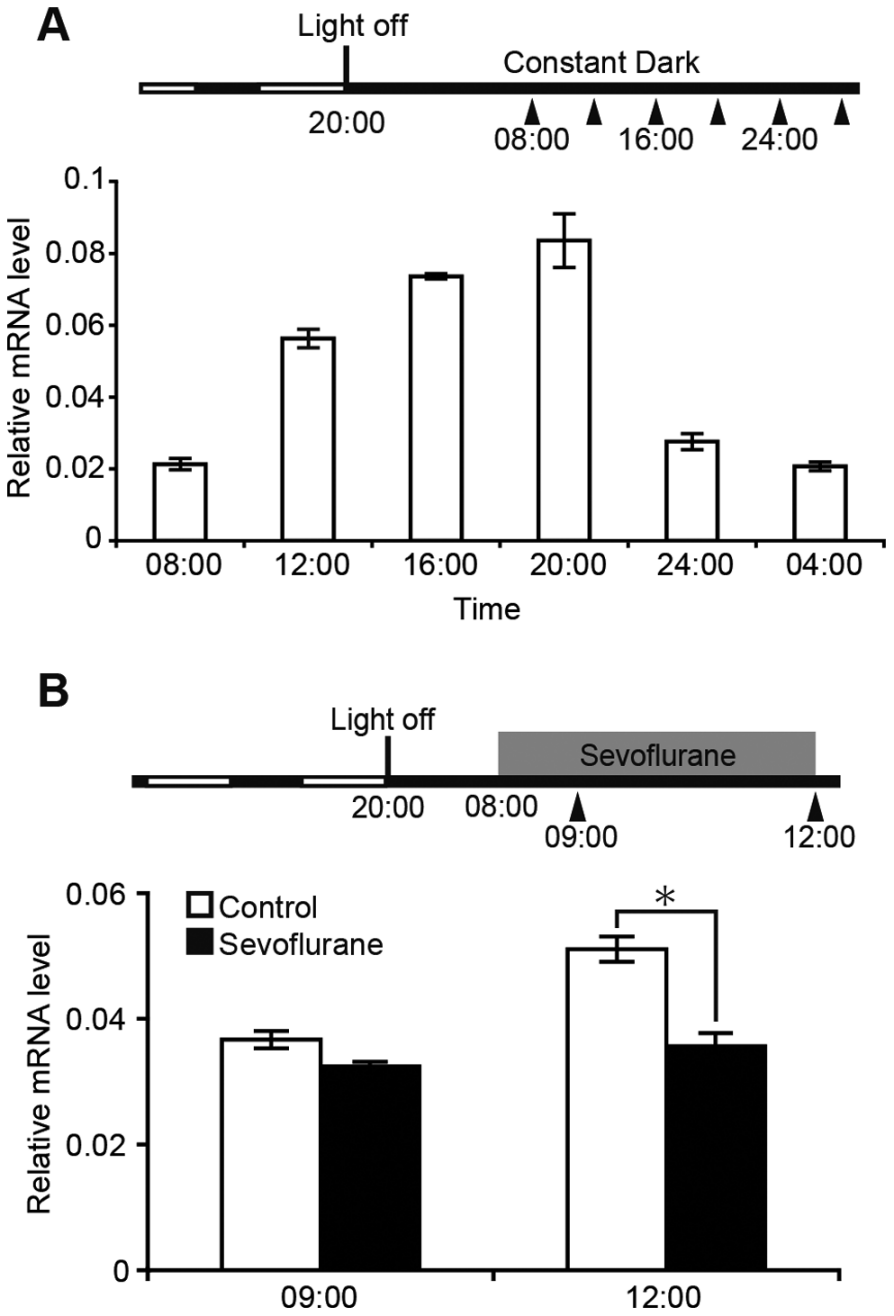
Inhibition of *Per2* expression in the SCN under sevoflurane anesthesia. (A) The light/dark conditions and sampling periods are illustrated in the upper scheme. White and black bars indicate light and dark periods, respectively. Arrowheads indicate the time of sampling. Lower graph shows the quantitative diurnal change in *Per2* expression. (B) The light/dark condition, sampling periods, and time of anesthetic treatment (gray bar) are illustrated in the upper scheme. Changes in *Per2* expression under anesthetic treatment are shown in the lower graph. Data are mean ± SEM. * denotes a statistically significant difference between anesthetic treatment and control (two-way ANOVA; *p*<0.05).

Throughout one sampling day, a clear circadian pattern was observed in the expression of the *Per2* gene in the SCN of non-anesthetized mice (Fig. 1A). *Per2* expression increased from early morning until evening, at which point it peaked and subsequently decreased from night until early morning. Sevoflurane anesthesia for 4 h (sampled at 12∶00) significantly suppressed *Per2* expression in the SCN compared with controls, whereas 1 h application did not (Fig. 1B). The *Per2* expression profile and its suppression by sevoflurane are consistent with our previous studies using quantitative *in situ* hybridization [7], [15], which validates and verifies our SCN sampling.

### Sevoflurane does not Affect the Methylation Profile of the *Per2* Promoter Region

**Figure 2:**
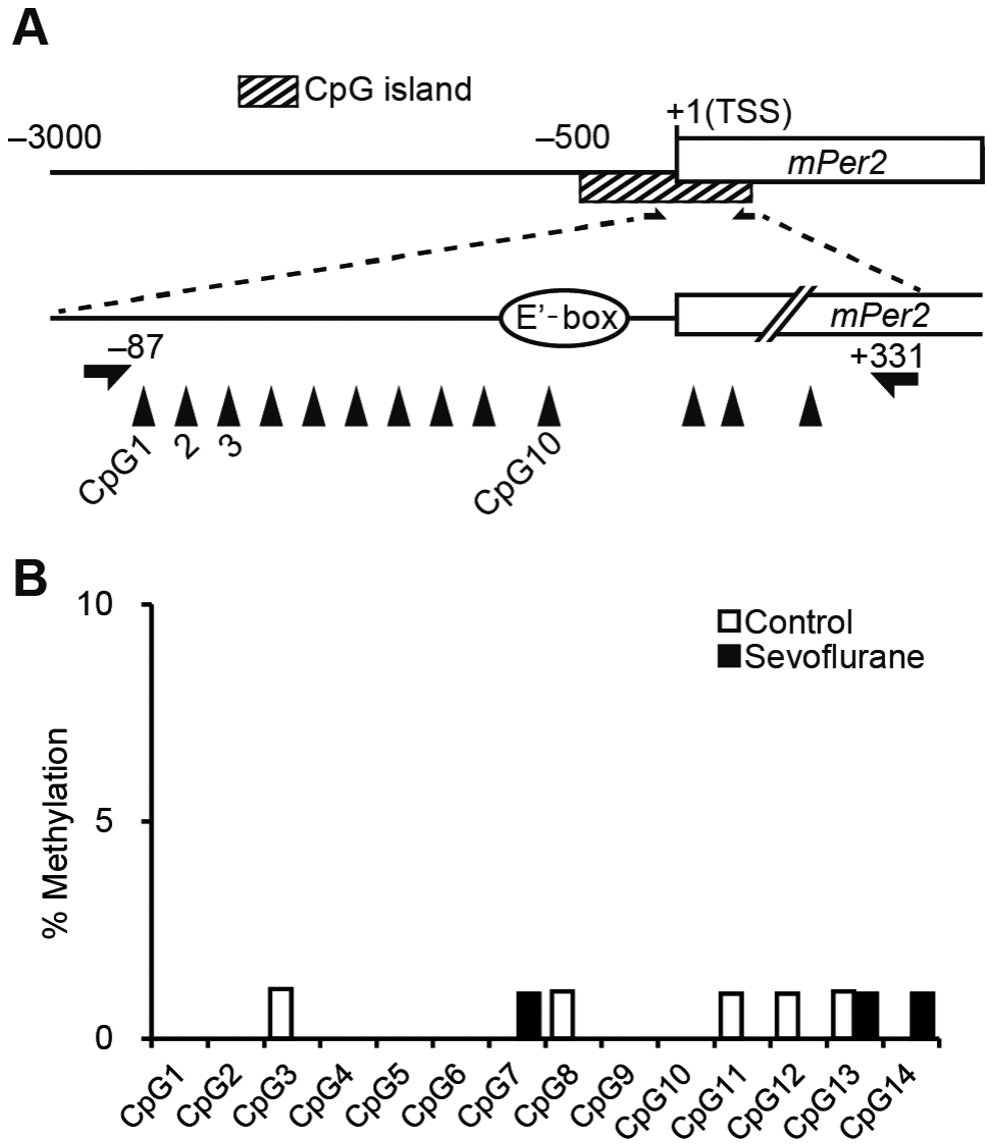
DNA methylation profile of the *Per2* promoter region following sevoflurane anesthesia. (A) Diagram of CpG island around the *Per2* locus. CpG sites are numbered and indicated by upward arrowheads. Arrows indicate pairs of methylation-specific primers used to amplify the region of the CpG island containing the E’-box. (B) Methylation rate of 10 individual sites in the *Per2* promoter and four CpG sites in exon 1.

We analyzed the *Per2* promoter region from −3000 bp of the transcription start site (TSS) to identify CpG islands according to the criteria proposed by Gardiner-Garden *et al*. [17] (length >200 bp, CG% >50, CpG observed/estimated >0.6). A CpG island spanning from −476 to +343 bp of the *Per2* TSS was found, which contains the E’-box sequence (CACGTT) [18], but not the CRE sequence [19]. We designed a pair of methylation-specific primers to amplify the region containing the E’-box (−87/+331 from the TSS) (Fig. 2A). The *Per2* promoter region assessed in this study was in a hypomethylated state. There was no significant change in the methylation profile of the sequence around the E’-box in the *Per2* promoter region under anesthetic treatment (Fig. 2B).

### Sevoflurane Suppresses Histone Acetylation in the Proximal Region of the *Per2* Promoter

**Figure 3:**
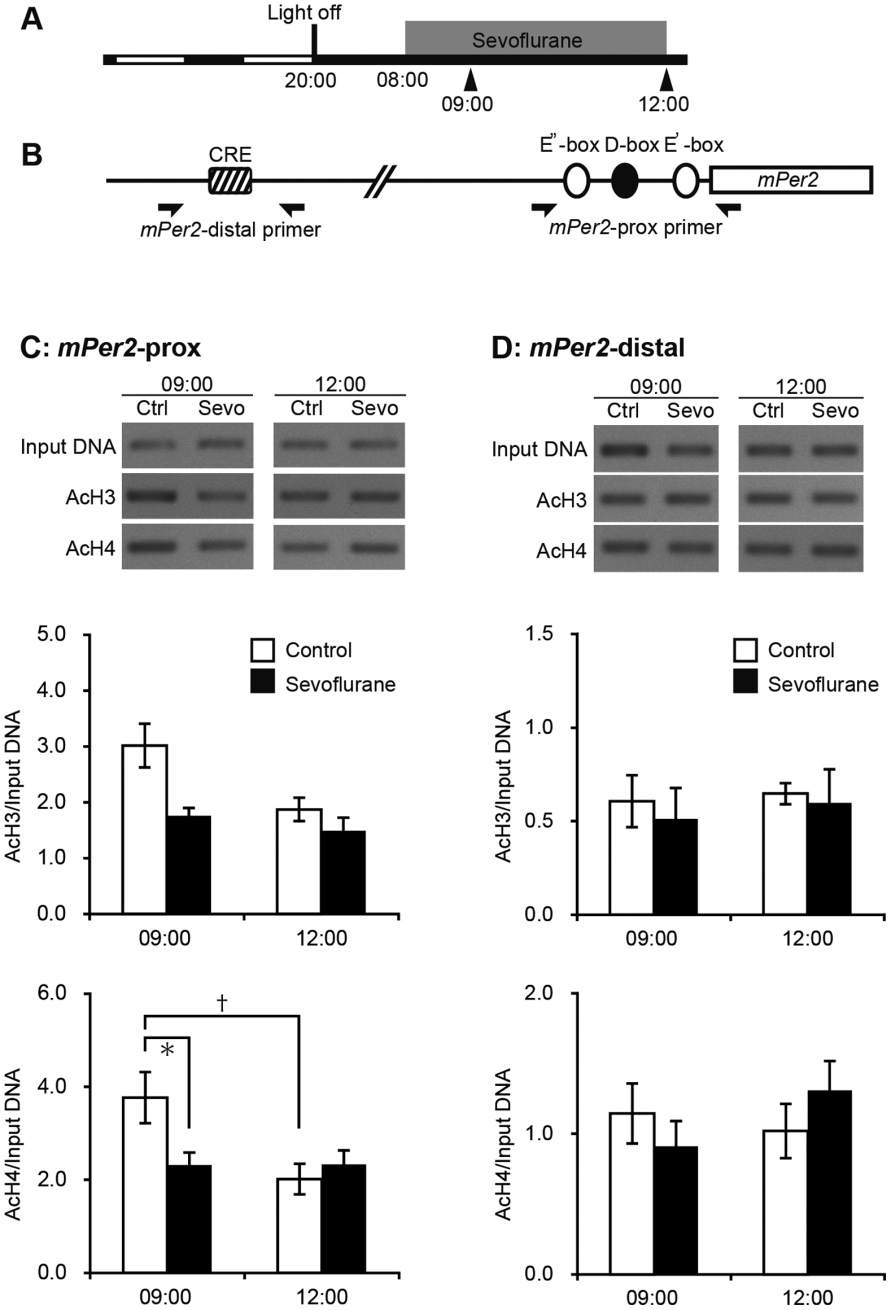
Changes in histone acetylation at CRE and E’-box in the *Per2* promoter under sevoflurane anesthesia. (A) Schematic representation of the light/dark condition, sampling periods, and time of anesthetic treatment (gray bar). Arrowheads indicate the time of sampling. (B) Schematic representation of *Per2* promoter. The positions of primer pairs used for PCR are indicated by arrows. (C, D) Representative PCR results and acetylation levels of histone H3 or H4 at the proximal (C) or distal (D) regions of the *Per2* promoter. Data are mean ± SEM. * denotes a statistically significant difference between control and anesthetic treatment. † denotes a statistically significant difference between the different time periods of anesthetic exposure (two-way ANOVA followed by Bonferroni test; *p*<0.05).

We used ChIP assays to examine the effect of sevoflurane on the acetylation status of histones H3 and H4 in the *Per2* promoter region. Two-way ANOVA revealed significant main effects of sevoflurane treatment on the acetylation of histone H4 in the E’-box region, and a significant interaction between time and sevoflurane treatment. Histone H4 acetylation in the E’-box of the *Per2* promoter was significantly suppressed by sevoflurane treatment for 1 h (sampled at 09∶00), but not by treatment for 4 h (sampled at 12∶00), compared with untreated controls (Fig. 3). Two-way ANOVA also revealed a significant main effect of sevoflurane treatment on the acetylation of histone H3 in the E’-box region, but there was no significant interaction between time and sevoflurane treatment (Fig. 3C). Sevoflurane did not significantly affect histone H4 and H3 acetylation in the distal region (Fig. 3D).

### Sevoflurane Suppresses Binding of CLOCK/BMAL1 Complex to the *Per2* E’-box Region

**Figure 4:**
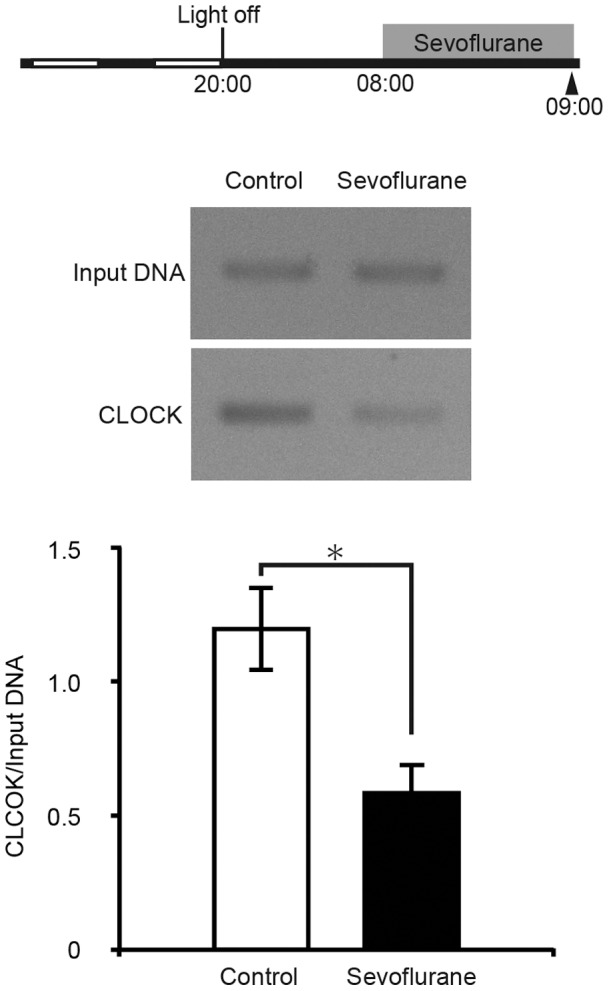
Suppression of CLOCK binding to the E’-box in the *Per2* promoter region under sevoflurane anesthesia. The light/dark condition, sampling periods, and time of anesthetic treatment (gray bar) are illustrated in the upper scheme. Arrowheads indicate the time of sampling. Representative PCR results are shown in the middle panel. CLOCK-E’-box binding in each sample is shown in the lower graph. Data are mean ± SEM. * denotes a statistically significant difference between control and anesthetic treatment (Student’s t-test; *p*<0.05).

To test the hypothesis that histone H4 acetylation is suppressed *via* the inhibition of CLOCK/BMAL1 binding to the E’-box, we measured CLOCK binding to this motif using a ChIP assay with an anti-CLOCK antibody. Anesthesia with sevoflurane for 1 h significantly decreased the binding of CLOCK to the E’-box compared with non-treated mice (Fig. 4).

### Sevoflurane does not Affect NAD^+^ Levels

**Figure 5:**
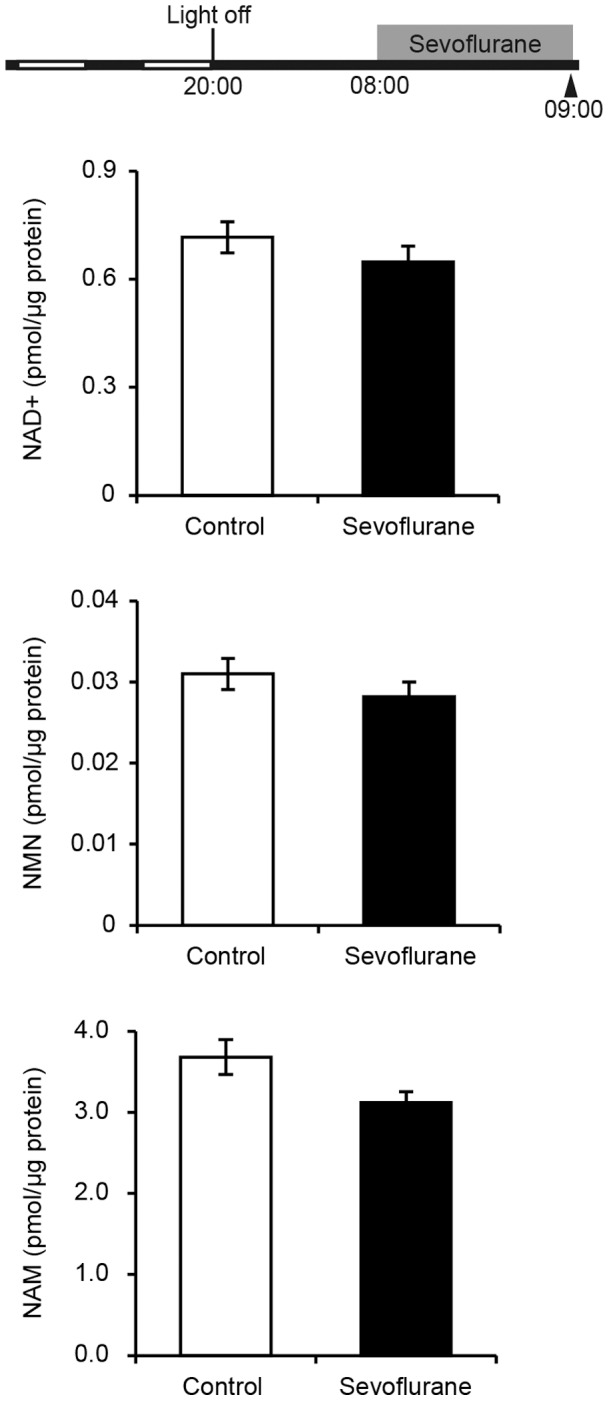
Effects of sevoflurane treatment on NAD+, NMN, and NAM levels in the SCN. Data were normalized using estimations of the total protein in each sample, and are shown as mean ± SEM (Student’s t-test; *p*<0.05).

To determine whether the reduced CLOCK binding to the E’-box is a consequence of an increase in NAD^+^ and subsequent SIRT1 activation, we assessed NAD^+^ and its metabolites after 1 h sevoflurane treatment. A salvage pathway comprising NAD^+^, NAM, and NMN is reported to be critical in regulating intracellular NAD^+^ [20]. Although NAM inhibits SIRT1 activity, NAD^+^ activates SIRT1. NAD^+^, NMN, and NAM concentrations were unchanged between control mice and mice treated with sevoflurane for 1 h (Fig. 5).

## Discussion

We previously reported that sevoflurane treatment reversibly suppressed *Per2* expression in the SCN [7]. In this study, we aimed to elucidate the molecular mechanism of this suppression. Our previous *in vitro* study using *Per2-dLuc* transgenic rats showed that sevoflurane suppressed mouse *Per2* promoter-driven luciferase expression, indicating that it acts by modulating the *Per2* promoter [8]. We focused here on the epigenetic events occurring in the *Per2* promoter in the SCN under anesthesia because it is well known that *Per2* expression is controlled by the balance between histone acetylation and deacetylation [11], [14]. It was assumed that DNA in the *Per2* promoter region, or histones associated with this region, could be epigenetically modified by sevoflurane treatment, so we performed methylation assays on the *Per2* promoter region and ChIP assays on the histones around which the *Per2* promoter region is winded.

We divided the promoter into two regions: a proximal region regulated by the acetyltransferase heterodimer CLOCK/BMAL1 and a distal region regulated by cAMP-dependent signaling [11], [21]. The proximal promoter region contains the E’-box, E’’-box, and D-box and is indispensable for regulation of *Per2* transcription. The E’-box located at −24 to −19 is essential in generating oscillations in *Per2* expression *in vivo* [18] and *in vitro* [22]. The E’’-box located at −162 to −157 is involved in the phase delay of *Per2* oscillation. The D-box (−152 to −145) is also required for robust circadian expression of *Per2* [23]. The E’- and E’’-boxes are activated by CLOCK/BMAL1 [24]. In this study, we found no effect of anesthesia on the methylation status of the *Per2*-promoter region, although *Per2* promoter region methylation has been linked to obesity and metabolic syndrome [25]. In contrast, the ChIP assays revealed reduced histone H4 acetylation in the proximal promoter region under anesthesia compared with that in non-treated mice. This decrease was observed prior to the significant drop in *Per2* expression. There are several literatures reporting the time lag between histone acetylation and clock gene expression [11], [26]. These time lags might be reflecting the time needed for changes in *Per2* transcript to be detected after alteration in histone acetylation. The ChIP assay using an anti-CLOCK antibody also revealed a decrease in CLOCK binding to the proximal region of the promoter under anesthesia. These results suggest that sevoflurane anesthesia diminishes CLOCK binding to the E’- and/or E”-boxes, leading to a decreased histone acetylation, and result in suppression of *Per2* expression. CRE binding proteins induce CRE-mediated gene transcription in response to changes in cAMP levels, and studies with CRE-mutated mice have shown that this mechanism is indispensable for the transcriptional oscillation of the *Per2* gene [19]. In this study, we detected no changes in histone acetylation associated with the CRE sequence under anesthetic treatment, indicating that sevoflurane suppresses histone acetylation associated with the E’- and E”-boxes but not that associated with CRE, in the circadian expression of *Per2*. On the other hand, the light-induced *Per2* expression is mediated by CRE [21] and light-induced *Per2* expression is suppressed by sevoflurane [7]. Thus, the epigenetic modification of CRE in the *Per2* promoter may contribute to the effects of sevoflurane on light-induced *Per2* expression.

Several other clock genes, including *Per1*, *Cry1*, *DEC1*, *DEC2*, *RevErbAa*, *RevErbAb*, and *RORc*, are regulated *via* the E/E’ box [27], so it is likely that sevoflurane inhibition of CLOCK binding to the E/E’ box could affect these genes also and lead to phase delay in circadian rhythms in the SCN. The effects of anesthetics on the expression of clock genes other than *Per2* remain to be elucidated.

The CLOCK/BMAL1 complex is assembled in conjunction with the NAD^+^-dependent histone deacetylase, SIRT1. Binding of the CLOCK/BMAL1/SIRT1 complex to the E-box is therefore regulated by NAD^+^, and this complex dissociates from the E-box at high NAD^+^ concentrations, which leads to histone deacetylation [14]. We strictly dissected the SCN from the brain slices to ensure accurate measurement of NAD^+^ and its metabolites in this region and found that 1 h of sevoflurane treatment induced no effect on the levels of NAD^+^ and its metabolites in the SCN. These results indicate that dissociation of CLOCK from the E’ or E”-box in the *Per2* promoter over this time period is not due to an increase in NAD^+^. In mammals, nicotinamide phosphoribosyltransferase (NAMPT) initiates the primary NAD^+^ biosynthesis pathway by converting NAM and 50-phosphoribosyl-1-pyrophosphate (50-PRPP) to NMN, which is the rate-limiting step in this NAD^+^ biosynthesis [28], [29]. NAD^+^ synthesis in the SCN may be dependent on NMN derived from the immediate vicinity because of the very low expression levels of intracellular NAMPT in the neurons [30]. Our recent *in vitro* study showed that sevoflurane treatment decreased *Per2* promoter activity in isolated SCN tissues when NMN replenishment from the surroundings was restricted [8]. These results from our *in vitro* study and the NAD^+^ quantification in this study suggest that suppression of CLOCK/BMAL1 activity is not due to any increase in NAD^+^. It has been reported that the CLOCK/BMAL1 and related NPAS2/BMAL1 heterodimeric acetyltransferase complexes are regulated by the redox state of NAD^+^ cofactors. The reduced forms of these redox cofactors, NADH and NADPH, strongly enhance the DNA binding of the acetyltransferase complex, whereas the oxidized forms inhibit binding [31]. In the cytoplasm, NADH content is ∼0.1% that of NAD^+^. Thus, even if the redox state of NAD^+^ cofactors is modified, the NAD^+^ content should be almost unchanged. Sevoflurane treatment might affect the binding activity of acetyltransferase complexes *via* the redox state of NAD^+^ cofactors, but did not do so by any modulation of NAD^+^ content. Further investigation is necessary to understand these systems.

Neither the direct target of sevoflurane nor its effect on intracellular signaling is yet known. *In vitro* studies revealed that tetrodotoxin drastically reduced *Per2* and *Per1* expression, suggesting that intercellular communication associated with electrophysiological events could be necessary for *Per1* and *Per2* expression [32], [33]. Most SCN neurons are GABAergic and express GABA_A_ ionotropic receptors [34]–[37], the activation of which suppresses *Per1* and *Per2* expression in the SCN [38], [39]. Taken together with the report that sevoflurane affects the GABA_A_ receptor [40], [41], it is possible that sevoflurane could induce modification of SCN GABA_A_ receptors and thus affect CLOCK binding to the E’- or E”-box *via* an intracellular signaling system. It has been reported that ketamine, another anesthetic with NMDA antagonist activity, influences the function of the circadian molecular machinery. ChIP analyses revealed that ketamine alters the recruitment of the CLOCK/BMAL1 complexes to circadian gene promoters [42]. It is possible that sevoflurane and ketamine use common signaling pathways to inhibit CLOCK activity.

In summary, we found that sevoflurane-induced epigenetic events underlie the suppression of *Per2* expression and may also affect the expression of various other circadian genes. The relationships between these epigenetic events and unconsciousness, reduced pain perception, and post-surgical morbidity are of great importance. We believe that better understanding of the effects of anesthetics on intracellular signaling and gene transcription in the brain is crucial to make improvements in the safety of anesthesia, and that our present study contributes significantly to this understanding.

## Supporting Information

Figure S1 **The dissected SCN sample. Brains of mice were cut into 300 µm thick slices using a cryostat.** A white arrow shows the dissected SCN using a microdissection punch (φ 1.0 mm) at −20°C under a stereomicroscope.
(TIF)

Figure S2 **Expression of** ***Hsp90ab1*** **after 4 h sevoflurane treatment.** The expression of the *Hsp90ab1* in the SCN of sevoflurane-treated (treated for 4 h, from 08∶00 to 12∶00) and control mice were quantified using real time PCR. Data are mean ± SEM. No significant change was observed between sevoflurane-treated mice and control mice.
(TIF)

Figure S3 **Verification of the bisulfite reaction.** Bisulfite conversion of the samples was performed in parallel with the conversion of control DNA supplied in the MethylEasy Xceed Kit. Bisulfite conversion was verified by PCR using primers used in this study (A and B, 320 base pair), and control primers supplied in the kit (C, D, and nc, 240 base pair). M; marker, A; bisulfite-treated DNA from non-anesthetized mice, B; non-bisulfite-treated DNA from non-anesthetized mice, C; bisulfite-treated control, D; non-bisulfite-treated control, nc; no DNA.
(TIF)

Text S1 **LC-MS quantification of NAD+ and its metabolites.**
(DOCX)
