# Supplementary figures and images for: Epigenetic Suppression of Mouse Per2 Expression in the Suprachiasmatic Nucleus by the Inhalational Anesthetic, Sevoflurane
Source: PLoS One. 2014 Jan 31;9(1):e87319. doi: 10.1371/journal.pone.0087319 (PMC3909093; doi:10.1371/journal.pone.0087319)

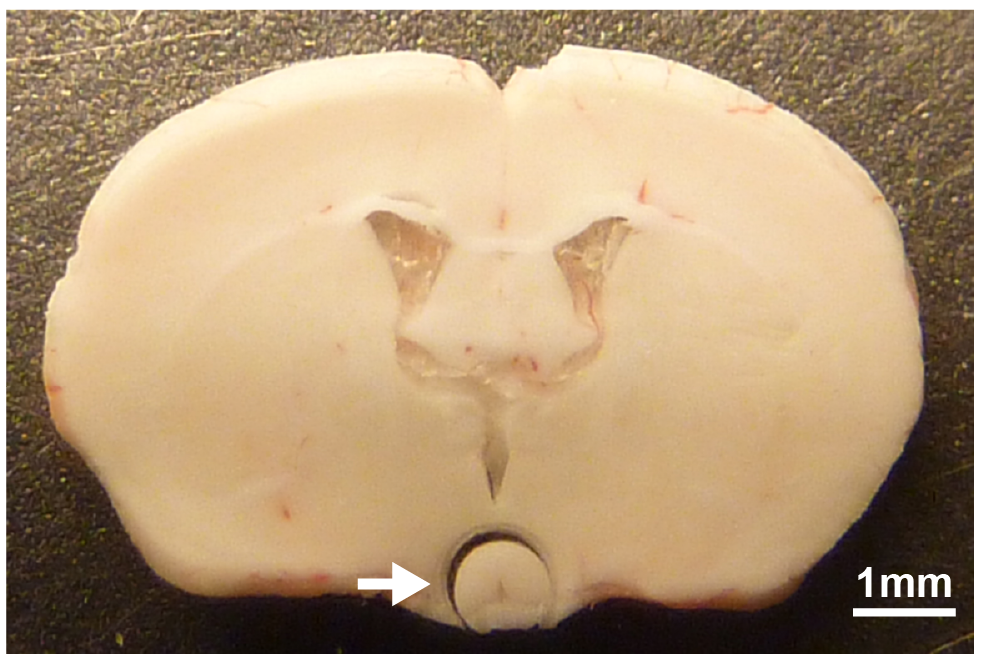

Supplement: Figure S1 — The dissected SCN sample. Brains of mice were cut into 300 µm thick slices using a cryostat. A white arrow shows the dissected SCN using a microdissection punch (φ 1.0 mm) at −20°C under a stereomicroscope. (TIF) [file pone.0087319.s001.tif]

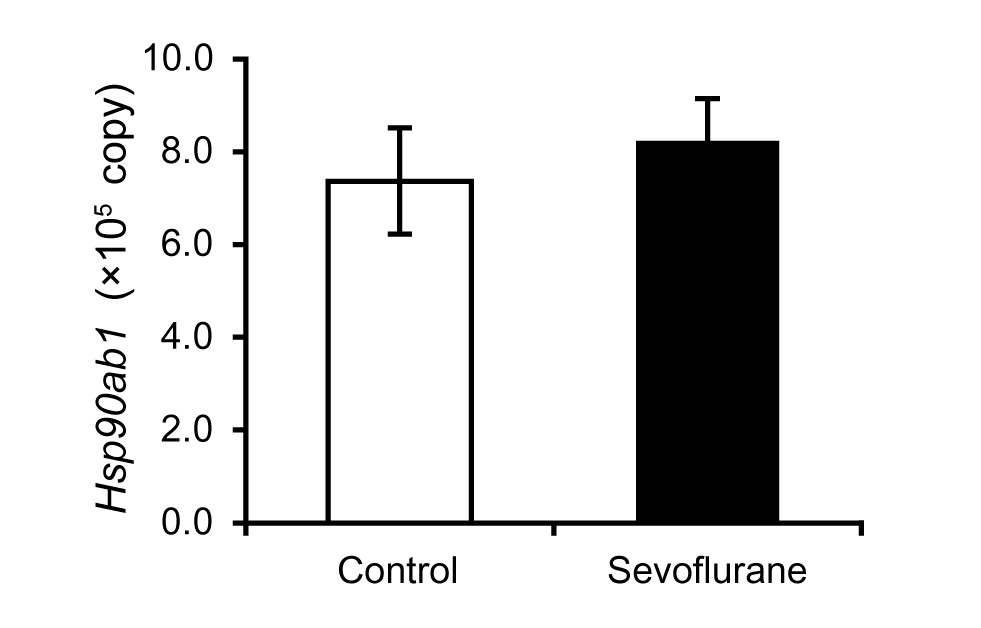

Supplement: Figure S2 — Expression of Hsp90ab1 after 4 h sevoflurane treatment. The expression of the Hsp90ab1 in the SCN of sevoflurane-treated (treated for 4 h, from 08∶00 to 12∶00) and control mice were quantified using real time PCR. Data are mean ± SEM. No significant change was observed between sevoflurane-treated mice and control mice. (TIF) [file pone.0087319.s002.tif]

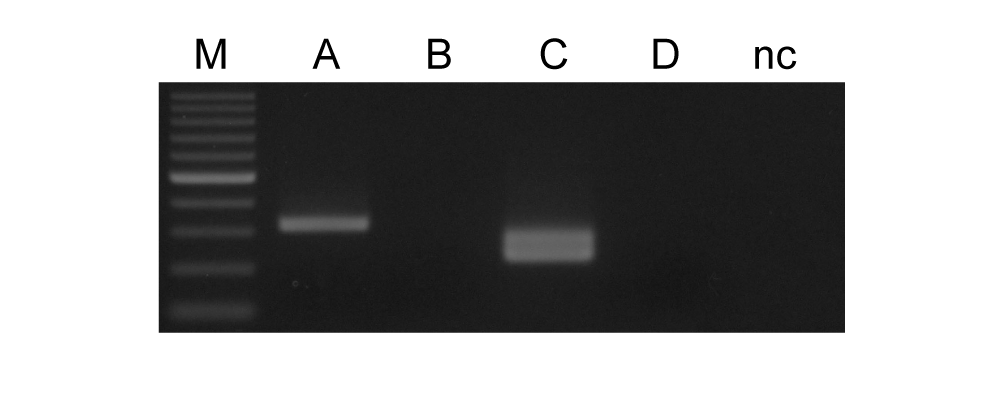

Supplement: Figure S3 — Verification of the bisulfite reaction. Bisulfite conversion of the samples was performed in parallel with the conversion of control DNA supplied in the MethylEasy Xceed Kit. Bisulfite conversion was verified by PCR using primers used in this study (A and B, 320 base pair), and control primers supplied in the kit (C, D, and nc, 240 base pair). M; marker, A; bisulfite-treated DNA from non-anesthetized mice, B; non-bisulfite-treated DNA from non-anesthetized mice, C; bisulfite-treated control, D; non-bisulfite-treated control, nc; no DNA. (TIF) [file pone.0087319.s003.tif]
