# Supplementary material for: Epigenetic Suppression of Mouse Per2 Expression in the Suprachiasmatic Nucleus by the Inhalational Anesthetic, Sevoflurane
Source: PLoS One. 2014 Jan 31;9(1):e87319. doi: 10.1371/journal.pone.0087319 (PMC3909093; doi:10.1371/journal.pone.0087319)
Supplement: Text S1 — LC-MS quantification of NAD+ and its metabolites. (DOCX) [file pone.0087319.s004.docx]

**Supplemental information**

*LC-MS quantification of NAD^+^ and its metabolites*

Dissected SCN were dispersed by trituration in 0.1 mL of 0.5 N perchloric acid, stored on ice for 10 min, and then centrifuged at 15,000×g for 10 min. The amounts of the NAD-related compounds in the supernatants were quantified using an LC-MS system consisting of a reverse-phase LC (Agilent 1200; Agilent Technologies, Santa Clara, CA, USA) coupled with an Agilent 6410 triple-stage quadrupole mass spectrometer (Agilent Technologies), operated with electrospray ionization in the positive-ion mode. Simultaneous detection of the NAD^+^ metabolites was performed in multiple reaction monitoring (MRM) mode.

The LC chromatographic separation was performed on a ZORBAX Eclipse XDB-C18 column (4.6×150 mm, 5 μm). The mobile phase comprised two solvents: solvent A (5 mM ammonium acetate) and solvent B (acetonitrile) and was delivered at a flow rate of 0.2 mL/min. Total run time was 25 min. Initial conditions involved a mixture of solvent A and B at a ratio of 90:10 v/v. One minute after sample injection, the solvent ratio was modulated linearly over 4 min to 50:50, then maintained at this level for 7 min. Solvent ratio was then further modulated to 100% solvent B over 0.5 min, then maintained at this level for 2 min. Finally, the solvent ratio was returned to the initial conditions (90:10 v/v solvent A:solvent B) over 0.5 min and maintained at this level for the final 10 min of each run.

The ionization conditions for LC-separated compounds were as follows: ion spray voltage, 4000 V; drying gas flow rate, 10 L/min; drying gas temperature, 350 °C; nebulizer gas, 70 psi. MRM parameters were as follows: collision energy, 25V; fragmenter voltage, 100 V. Nitrogen was used as the collision gas in the collision cell. The instrument monitored the m/z transition, from 664 to 428 for NAD, from 124 to 80 for NAM, and from 335 to 123 for NMN. Here, m and z represent the mass and charge of a compound, respectively. Calibration curves constructed using standard compounds (Sigma N1636 for NAD, Sigma 47865-U for NAM, and Sigma N3501 for NMN, respectively) showed that the curves were linear between 1 and 1500 pg on the column for each compound.
